# Treatment Modality for Stage IB Peripheral Non-Small Cell Lung Cancer With Visceral Pleural Invasion and ≤3 cm in Size

**DOI:** 10.3389/fonc.2022.830470

**Published:** 2022-02-18

**Authors:** Weijia Huang, Han-Yu Deng, Ming-Ying Lin, Kai Xu, Yu-Xiao Zhang, Chi Yuan, Qinghua Zhou

**Affiliations:** ^1^ Lung Cancer Center, West China Hospital, Sichuan University, Chengdu, China; ^2^ West China School of Medicine, Sichuan University, Chengdu, China

**Keywords:** non-small cell lung cancer, visceral pleural invasion, surgery, adjuvant chemotherapy, small-sized

## Abstract

**Purpose:**

To compare the survival difference among lobectomy, segmentectomy, and wedge resection and investigate the role of adjuvant chemotherapy for early-stage small-sized non-small cell lung cancer (NSCLC) with visceral pleural invasion (VPI).

**Methods:**

Patients diagnosed with stage IB peripheral NSCLC with VPI and ≤3 cm in size in the Surveillance, Epidemiology, and End Results database between 2004 and 2015 were included, and the pleural layer (PL) invasion status was identified to recognize the tumors with VPI, including PL1 and PL2. We conducted Cox proportional hazards model in multivariable analysis and subgroup analysis *via* propensity score matching (PSM) method and Cox regression method to figure out the optimal therapy for these patients.

**Results:**

A total of 1,993 patients were included, all of whom received surgery, and the median follow-up was 33 months (range, 1–83 months). In multivariable analysis, age, gender, histology, pathological grade, lymph node examination, surgical approaches, and radiotherapy were independent prognostic factors for overall survival (OS). Lobectomy was superior to sublobar resection [hazard ratio (HR) = 1.41; 95% CI, 1.08–1.83], and wedge resection was associated with impaired survival compared to lobectomy (HR = 1.64; 95% CI, 1.22–2.20) in PSM analyses. In subgroup analysis, lobectomy was superior to sublobar resection among those aged <70 years (HR = 1.81; 95% CI, 1.13–2.90), female (HR = 1.75; 95% CI, 1.21–2.53), and 1–20 mm in size (HR = 1.61; 95% CI, 1.11–2.33). No survival benefit was observed for adjuvant chemotherapy.

**Conclusions:**

Lobectomy was superior to wedge resection and comparable with segmentectomy for stage IB NSCLC (≤3 cm) with VPI, and adjuvant chemotherapy could not benefit these patients, even in those with sublobar resection. The preferred surgical procedure remains to be studied in prospective controlled trials.

## Introduction

Visceral pleural invasion (VPI) was announced as the poor prognostic factor for early-stage non-small cell lung cancer (NSCLC), and previous research indicated that the T category of TNM classification would be further evaluated by VPI extent (1–4). A tumor ≤3 cm in size with VPI and lymph node negative would be upstaged to T2, even though a tumor 3–5 cm in size without other clinicopathological characteristics specified was still T2 disease in the eighth edition of TNM classification (4). VPI could be identified on conventional CT images by pleural tags preoperatively that might increase the accuracy of early diagnosis of VPI (5). A population-based study carried out between 1989 and 2003 by the California Cancer Registry, including 10,545 patients with stage IB NSCLC, announced that around 20% of patients were classified as stage IB resulting from VPI, hilar atelectasis, or obstructive pneumonitis, even though they were ≤3 cm in size (6). Modified Hammar Classification suggested that a tumor invading beneath the elastic layer was referred to as pleural layer 0 (PL0), PL1 as invading beyond the elastic layer, PL2 as invading the pleural surface, and PL3 as invading the parietal pleura, among which PL1 and PL2 were T2 descriptors (7, 8). While prior research indicated that the adverse effect of VPI might be mainly distributed in NSCLC with N0 disease and 1–3 cm in size, the additional effect of invasiveness on VPI was found weakened with N stage upstaging and tumor size increasing (9). Furthermore, a multicenter retrospective study investigated 639 patients with completely resected NSCLC and found that the survival difference in N0 disease was only observed between PL0 and PL1 (P = 0.003) but not between PL1 and PL2 (P = 0.97) (10). Therefore, the role of VPI in small-sized early-stage NSCLC needs to be established further.

Surgical resection with lymph node dissection was the recommended standard treatment for early-stage NSCLC, and adjuvant chemotherapy might be considered for operable stage IB NSCLC postoperatively, especially when patients were identified with several high-risk clinicopathologic characteristics, including large tumor size (>4 cm), VPI, lymphovascular invasion (LVI), and high-grade histology (4, 11–13). Current randomized controlled trials seldom evaluated the surgical approaches for node-negative NSCLC with VPI, and the preference between lobectomy and sublobar resection has not been determined (14, 15). In the subgroup analysis of the newly published systematic review, including 8,447 patients from 34 trials, an improved 5-year OS from 55% to 60% was noticed resulting from adjuvant chemotherapy in stage IB NSCLC (16). However, there was no significant survival benefit for adjuvant chemotherapy observed among stage IB patients in a large pooled analysis (HR = 0.93; 95% CI, 0.78–1.10) and majority of the randomized controlled trials but effective in stage II and IIIA patients or those with lymph node positive or a larger tumor size (17–20). Moreover, the stage IB patients might be impaired in survival when receiving adjuvant chemotherapy compared with postoperative observation (P = 0.021) (21). Nevertheless, Strauss et al. (22) suggested that adjuvant chemotherapy was potentially effective in stage IB malignancy among those with large tumor size (>4 cm).

Although the aggressiveness of VPI has been widely studied, the favorable treatment modality for small-sized node-negative NSCLC with VPI has not been described. The purpose of this study was to compare the survival difference among lobectomy, segmentectomy, and wedge resection and investigate the role of adjuvant chemotherapy for stage IB peripheral NSCLC with VPI and ≤3 cm in size *via* propensity score matching (PSM) method in the Surveillance, Epidemiology, and End Results (SEER) database, which has been operated since 1973 by the National Cancer Institute.

## Methods

### Patient Selection and Data Extraction

**Figure 1:**
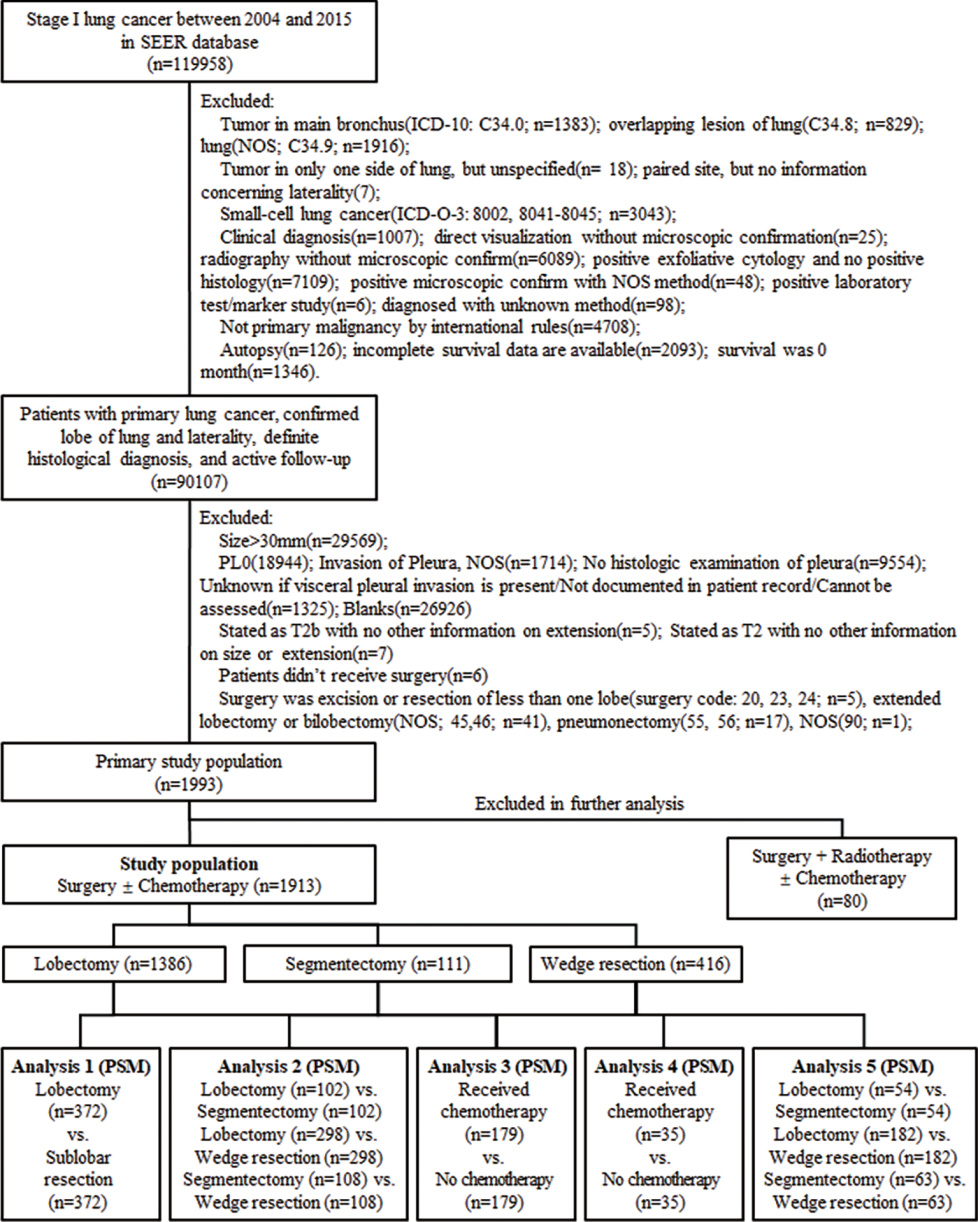
The process of data extraction from the Surveillance, Epidemiology, and End Results (SEER) database and the following propensity score matching (PSM) analyses. ICD-10, International Classification of Diseases, Tenth Revision; ICD-O-3, International Classification of Disease for Oncology, Third Edition; NOS, not otherwise specified; vs., versus.

We identified the patients from the SEER database *via* SEER Stat (version 8.3.8; www.seer.cancer.gov) in February 2021 with the identifier 11151-Nov2019. This research was accorded with the amended Declaration of Helsinki, and consent from patients and research ethics approval were not required due to the data anonymization in the SEER database and elimination of patient identification. Patients diagnosed with stage IB peripheral NSCLC with VPI and ≤3 cm in size between 2004 and 2015 were included, and the inclusion and exclusion criteria were shown in a flowchart (**Figure 1**).

### Data Curation and Study Variables

Demographics, baseline characteristics, and treatment modalities were extracted, including age, gender, race, marital status, primary site, laterality, the total number of *in situ* tumors, tumor size, histology, pathological grade, the status of lymph nodes examined, chemotherapy, and radiotherapy. Cancer staging was in terms of the seventh edition of the American Joint Committee on Cancer Staging Manual, and the histological classification was in accord with the third edition of the International Classification of Disease for Oncology. We excluded centrally located tumors and diligently identified peripheral malignancies. We identified the pleural layer (PL) invasion status (site-specific factor 2; code: PL1/2) in the SEER database to identify the tumors with VPI. Age was divided into two cohorts determined by the median value of the study population. Those who were still alive at the end of the follow-up were considered censored when conducting survival analysis.

### Statistical Analysis

Demographics and baseline characteristics were compared *via* χ^2^ or ANOVA test. Considering the potential prognostic heterogeneity of those with radiotherapy compared to those without, we eliminated the patients with radiotherapy in further survival analyses. We first conducted Kaplan–Meier analyses to determine the prognostic factors in the study cohort, and the variables with P-value <0.2 were admitted to the multivariable analysis. We identified independent prognostic factors *via* Cox proportional hazards model in multivariable analysis, which accorded with the assumption of proportional hazards. Then, we conducted five PSM analyses (**Figure 1**), which referred to lower potential bias for nonrandomized patient selection. We conducted the nearest-neighbor matching method and logistic regression in PSM analysis (one unit matched to one unit), and the caliper was set to 0.2. After matching, all these covariables were balanced in each subgroup analysis, including age, race, gender, marital status, the total number of tumors, tumor size, histology, pathological grade, the status of lymph nodes examined, and surgical procedures, which were also assessed by standardized mean difference. In subgroup analysis, sublobar resection was first investigated instead of segmentectomy and wedge resection. The relative hazard ratio (HR) in subgroup analysis was determined by univariable analysis. A P-value <0.05 was identified as statistical significance, and all statistical analyses were performed using R 3.6.1 (R Foundation for Statistical Computing, Vienna, Austria) and R packages (tableone, MatchIt, Hmisc, rms, survival, survminer).

## Results

A total of 1,993 patients were included in the primary study cohort with a median age of 70 years (range, 35–96 years), 80 (4.0%) of whom received radiotherapy. All patients received surgery, in which 1,420 (71.2%) received lobectomy, 116 (5.8%) received segmentectomy, and 457 (22.9%) received wedge resection (**e-Table 1**). In the primary study cohort, the median follow-up was 33 months (range, 1–83 months). The 1-, 3-, and 5-year overall survival (OS) rate was 92.8%, 73.9%, and 60.8%. As divided by surgical approaches, the 5-year OS rate of those with lobectomy, segmentectomy, and wedge resection was 66.0%, 51.8%, and 46.7%, respectively. Then, we eliminated the patients with radiotherapy from the preliminary study cohort, contributing to the exact study population, and conducted survival analyses.

In multivariable analysis, age, gender, histology, pathological grade, lymph node examination, and surgical approaches were independent prognostic factors with regard to OS (**Table 1**). Age over 70 years (HR = 1.75; 95% CI, 1.47–2.10; P < 0.001), male (HR = 1.29; 95% CI, 1.09–1.52; P = 0.003), 21–30 mm (HR = 1.19; 95% CI, 1.00–1.41; P = 0.048), squamous cell carcinoma or other histology types (HR = 1.31; 95% CI, 1.10–1.56; P = 0.002), and receiving wedge resection (HR = 1.31; 95% CI, 1.05–1.63; P = 0.017) were associated with poor OS.

**Table 1:** Univariable analysis and multivariable analysis of the study population regarding overall survival and cancer-specific survival.

Characteristics	Overall survival			Cancer-specific survival		
	P-Value	HR (95% CI)	P-Value	P-Value	HR (95% CI)	P-Value
**Age**	<0.001			0.002		
≤70 years		Ref.			Ref.	
>70 years		1.75 (1.47–2.10)	<0.001		1.33 (1.01–1.75)	0.039
**Gender**	0.001			0.348		
Female		Ref.				
Male		1.29 (1.09–1.52)	0.003			
**Tumor size**	0.110			0.043		
1–20 mm		Ref.			Ref.	
21–30 mm		1.19 (1.00–1.41)	0.048		1.36 (1.04–1.78)	0.023
**Histology**	<0.001			0.001		
AC		Ref.			Ref.	
SCC/Others		1.31 (1.10–1.56)	0.002		1.34 (1.02–1.76)	0.034
**Grade**	0.004			0.005		
I/II		Ref.			Ref.	
III/IV/UK		1.18 (0.99–1.40)	0.070		1.33 (1.01–1.74)	0.040
**LN examined**	<0.001			0.004		
No/UK		Ref.			Ref.	
Yes		0.63 (0.49–0.81)	<0.001		0.76 (0.50–1.14)	0.182
**Surgery**	<0.001			<0.001		
Lobectomy		Ref.			Ref.	
Segmentectomy		1.21 (0.85–1.73)	0.292		1.44 (0.85–2.45)	0.178
Wedge resection		1.31 (1.05–1.63)	0.016		1.41 (1.00–1.99)	0.049
**Chemotherapy**	0.027			0.085		
No/UK		Ref.			Ref.	
Yes		0.88 (0.64–1.22)	0.452		0.74 (0.43–1.26)	0.262

HR, hazard ratio; CI, confidence interval; Ref., reference; AC, adenocarcinoma; SCC, squamous cell carcinoma; UK, unknown; LN, lymph node.

**Figure 2:**
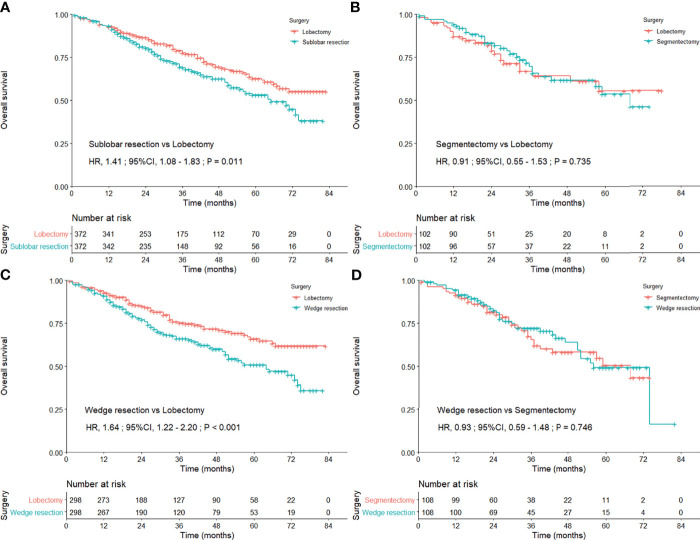
Kaplan–Meier curves comparing the overall survival after lobectomy and sublobar resection **(A)** lobectomy and segmentectomy **(B)** lobectomy and wedge resection **(C)** and segmentectomy and wedge resection **(D)** among the entire study population after propensity score matching.

We first compared the OS concerning surgical approaches between lobectomy and sublobar resection *via* PSM method, and lobectomy performed better (HR = 1.41; 95% CI, 1.08–1.83; P = 0.011; **Figure 2A**). In the comparisons of survival difference among these three surgical procedures (**e-Table 3**), only wedge resection was significantly inferior to lobectomy (HR = 1.64; 95% CI, 1.22–2.20; **Figure 2B**), and there was no statistical difference in the remaining (segmentectomy vs. lobectomy, P = 0.735; wedge resection vs. segmentectomy, P = 0.746; **Figures 2C, D** **)**. No positive findings could be concluded in further subgroup analyses with regard to the three surgical procedures (**e-Table 4**).

**Figure 3:**
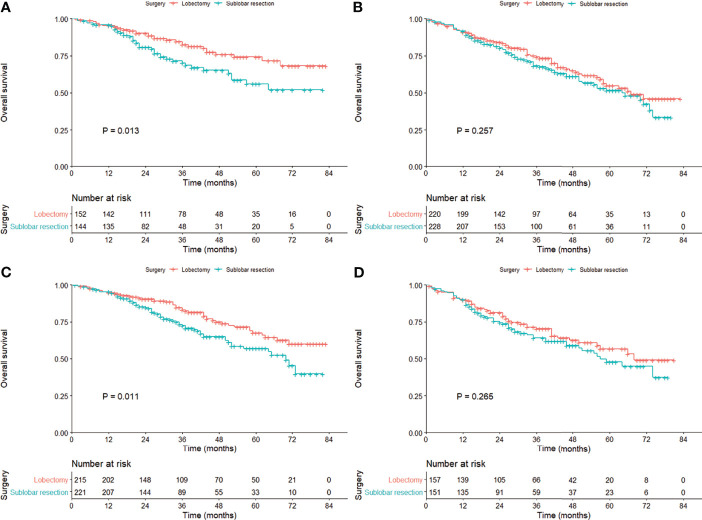
Kaplan–Meier curves comparing the overall survival between lobectomy and sublobar resection in the cohort subgrouped by age [**(A)** ≤70 years; **(B)**] >70 years) and tumor size [**(C)** 1–20 mm; **(D)**] 21–30 mm) after propensity score matching.

The OS of the patients treated with lobectomy was significantly superior to those treated with sublobar resection among those aged less than 70 years (HR = 1.81; 95% CI, 1.13–2.90), female (HR = 1.75; 95% CI, 1.21–2.53), and 1–20 mm in size (HR = 1.61; 95% CI, 1.11–2.33; **Table 2**, **Figure 3**). We also compared the three surgical approaches among those over 70 years in the subgroup analysis (**e-Table 5**), and even adjusting for propensity scores, we could not identify the favorable surgical approach that was superior in long-term survival (**e-Table 6**; all P-values >0.05).

**Table 2:** Subgroup analysis of surgical approaches and adjuvant chemotherapy in overall survival *via* Cox regression analysis after propensity score matching.

Characteristics	Sublobar resection vs. lobectomy		Chemotherapy, Yes vs. No/UK	
	HR (95% CI)	P-Value	HR (95% CI)	P-Value
**Age**				
≤70 years	1.81 (1.13–2.90)	0.014	0.92 (0.52–1.64)	0.784
>70 years	1.20 (0.87–1.65)	0.259	1.17 (0.58–2.34)	0.662
**Gender**				
Male	1.10 (0.76–1.61)	0.608	1.21 (0.57–2.57)	0.615
Female	1.75 (1.21–2.53)	0.003	0.91 (0.52–1.61)	0.755
**Tumor size**				
1–20 mm	1.61 (1.11–2.33)	0.012	1.78 (0.88–3.59)	0.110
21–30 mm	1.24 (0.85–1.80)	0.269	0.65 (0.36–1.20)	0.171
**Histology**				
AC	1.54 (1.09–2.19)	0.016	1.04 (0.60–1.80)	0.885
SCC/Other	1.24 (0.83–1.84)	0.292	0.91 (0.43–1.94)	0.807
**Grade**				
I/II	1.64 (1.14–2.35)	0.007	1.14 (0.62–2.09)	0.676
III/IV/UK	1.16 (0.79–1.71)	0.457	0.87 (0.45–1.68)	0.685
**Surgery**				
Lobectomy			0.99 (0.59–1.69)	0.982
Sublobar resection			1.09 (0.48–2.48)	0.830

vs., versus; HR, hazard ratio; CI, confidence interval; AC, adenocarcinoma; SCC, squamous cell carcinoma; UK, unknown.

**Figure 4:**
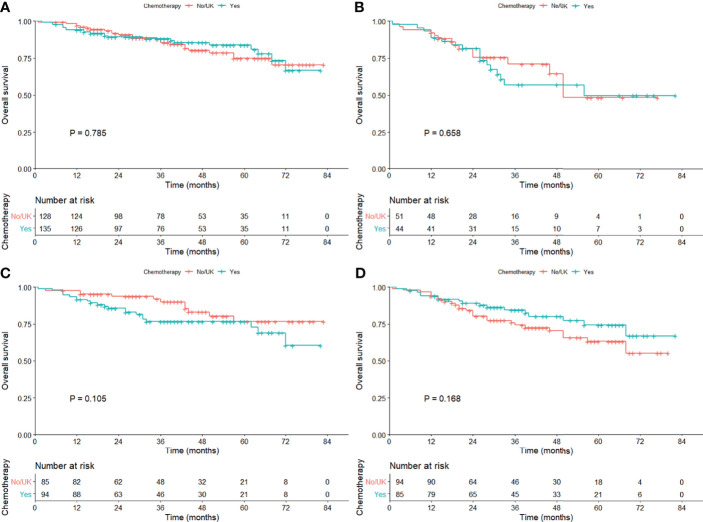
Kaplan–Meier curves comparing the overall survival among those with or without adjuvant chemotherapy in the cohort subgrouped by age [**(A)** ≤70 years; **(B)**] >70 years) and tumor size [**(C)** 1–20 mm; **(D)**] 21–30 mm) after propensity score matching.

To investigate the efficacy of adjuvant chemotherapy, we conducted PSM analysis in the exact study population and patients with sublobar resection in sequence, and the clinical characteristics were shown (**e-Tables 7, 8**). There was no survival benefit observed (**e-Figure 1**) and so as in subgroup analysis (**Table 2**). When stratified by tumor size, no statistical difference was observed (1–20 mm, P = 0.105; 21–30 mm, P = 0.168; **Figure 4**).

## Discussion

Lobectomy with mediastinal systematic lymph node dissection was the standard treatment for early-stage NSCLC, while sublobar resection was likely to be recommended in the small-sized malignancy considering the postoperative cardiopulmonary reserve. However, it remained confused as how to take the tumor size and VPI into account when making clinical decisions because VPI was considered as a poor prognostic factor in the small-sized NSCLC (≤3 cm) (2). We could identify the patients with possible VPI *via* conventional CT images by pleural tags (5), and thus, the result of preoperative VPI detection might be taken into consideration in preoperative conference on surgical procedures. Thus, we investigated the survival benefit from three surgical procedures and role of adjuvant chemotherapy for small-sized NSCLC with VPI. Lobectomy was likely to be superior to wedge resection and comparable to segmentectomy for stage IB NSCLC (≤3 cm) with VPI, and wedge resection was associated with impaired survival. Adjuvant chemotherapy might not improve the prognosis even in those who received sublobar resection.

Our results revealed that lobectomy might be associated with improved survival compared to sublobar resection, especially in tumors ≤20 mm, while lobectomy was comparable to segmentectomy, which was partly in line with the study by Schuchert et al. (14). Schuchert et al. (14) retrospectively reviewed 899 patients with stage I NSCLC with segmentectomy or lobectomy, in which the data were collected prospectively, and they found that lobectomy was superior to segmentectomy among stage IB patients with VPI (median OS, 29.6 vs. 22.7 months; P = 0.048). However, our findings were supposed to be interpreted with caution. Most of the sublobar resections were wedge resections that would skew the results of the cohort toward a poorer outcome. Besides, we could only identify the impaired survival from wedge resection when investigating these three surgical procedures in the subgroup analyses, with no other positive findings concluded. Moon et al. (15) found that survival was comparable between lobectomy and sublobar resection for stage I NSCLC ≤2 cm with VPI or LVI, and the dissected lymph node count might be responsible for the recurrence (HR = 0.914; 95% CI, 0.845–0.988). Hsu et al. (23) further indicated that more than 14 lymph node removement might be associated with survival improvement. We postulated that lobectomy was associated with more lymph node removement during surgery, and thus, survival benefit might result from the extensive intrapulmonary lymph node resection (24, 25). Secondly, lobectomy was favored in tumors no more than 20 mm but had not shown significant efficacy in tumors 20–30 mm. It might be referred to the inherent aggressiveness of the tumor ≤20 mm surpassing those with 20–30 mm in size that tumor could invade the visceral pleural layer when they were in a small size, and lobectomy might demonstrate improved survival for more aggressive malignancy compared with sublobar resection. The superior outcome of the lobectomy cohort in the smaller tumor group could also be explained by a greater proportion of wedge resections being performed for those tumors <2 cm, and more segmentectomies for those tumors >2 cm resulting in oncologic outcomes approximating lobectomy.

Furthermore, we could not conclude the favorable surgical approach in the elderly patients regarding long-term survival, which was in line with prior research (26), and might be attributed to a low malignant behavior of the tumor among the elderly compared with younger patients (27). Several studies claimed that the OS was not associated with the pathological stage in the elderly, and quite a few elderly patients might die of non-cancer-related causes, and thus, complete tumor resection was only a part in improving the prognosis (26, 28). Considering the preoperative comorbidities and postoperative complications of elderly patients, sublobar resection might be recommended among those over 70 years, which was in agreement with previous research (12, 29, 30).

In our research, adjuvant chemotherapy was not associated with improved survival even in those who received sublobar resection. A large population-based study was conducted between 2003 and 2006 *via* the National Cancer Database, which included 34,360 patients with T1-2N0M0 NSCLC, and no survival benefit was found among patients with ≤3 cm in size (31). In the study, they merely identified the efficacy of adjuvant chemotherapy grouped by tumor size, instead of subgrouping the small-sized tumors (≤3 cm) by the high-risk factors (including VPI), and thus, the potential beneficiary might be neglected. A large retrospective cohort study, including 50,814 patients with node-negative early-stage NSCLC, also indicated that survival benefit was not noticed in those who received chemotherapy with ≤3 cm in size (HR = 1.10; 95% CI, 0.96–1.26), while chemotherapy was associated with improved survival in 3–4 cm (only in those who received sublobar resection), 4–5 cm (VPI, LVI, or high-grade histology), and >5 cm (regardless of VPI, LVI, or high-grade histology) (13). However, a pooled analysis of systematic review, including six studies, found that the survival was comparable between tumor size ≤3 cm with VPI and 3–5 cm without VPI, and they suggested that stage IB NSCLC with 3–5 cm in size and VPI might be the candidate of adjuvant chemotherapy (2). Therefore, as claimed in our research and in agreement with current guideline (32), the role of adjuvant chemotherapy was still undefined in small-sized NSCLC with VPI, and there were several explanations for the negative findings regarding the efficacy of adjuvant chemotherapy, including the offset of survival benefit and adverse effect from chemotherapy, and the limited quantity of stage IB patients receiving chemotherapy. Our study helped to investigate the role of adjuvant chemotherapy in small-sized NSCLC with VPI, and it reminded that the potential beneficiaries of chemotherapy might be further subgrouped by other baseline characteristics that were not studied above, including performance status and pulmonary function.

Now that the statistical difference was limited when we evaluated the survival difference with respect to surgical approaches and adjuvant chemotherapy, some other clinicopathological characteristics might interfere with the survival benefit. Okada et al. (33) reviewed 498 node-negative NSCLC (227 pure-solid and 271 part-solid) with VPI and ≤3 cm in size, and they concluded that VPI was associated with poor survival in pure-solid tumors (HR = 2.129; 95% CI, 1.048–4.132), but not in part-solid tumors (HR = 0.925; 95% CI, 0.050–4.920). Considering VPI had a negative effect on pure-solid tumors, the association between VPI and solid components was likely to be further investigated. Liang et al. (34) conducted a retrospective study, including 1,055 resected NSCLC with elastic layer staining and found that the disease-free survival (DFS) and OS were comparable in tumors with either PL1 or PL0 (DFS, P = 0.468; OS, P = 0.388). They proposed that the tumors with ≤3 cm in size and PL1 were supposed to be defined as stage T1, and adjuvant chemotherapy might not improve the prognosis (34), which was in line with the previous study (35). Nevertheless, Kawase et al. (36) suggested that a significant survival difference was observed between PL0 and PL1, and PL1 and PL2, while Wo et al. (9) concluded that there was no significant difference in survival between PL1 and PL2. Qian classified the stage I lung adenocarcinoma into three risk stratifications, and they claimed that most patients pertained to be the intermediate-risk population proved by a prognostic model, including six clinicopathological characteristics (age, sex, tumor size, pathological subtype, VPI, LVI) (37). It might explain why no significant survival benefit was observed in those with adjuvant chemotherapy, and in this way, the definite clinicopathological characteristics for risk stratifications might be further investigated. However, with current available evidence, adjuvant chemotherapy may not be suggested for small-sized NSCLC with VPI.

There were several limitations in our research. Firstly, several variables were not available in the SEER database, which might lead to some bias in our conclusions, including performance status, pulmonary function, imaging data, and whether surgery was video-assisted thoracoscopic surgery. Secondly, due to the inherent insufficiency of a retrospective study, we could hardly perform a randomized selection of patients, for which we dedicated to balance the baseline characteristics *via* PSM analysis, and thus, we also tried to avoid making any definite recommendations about treatment modality.

## Conclusion

Lobectomy was likely to be superior to wedge resection and comparable to segmentectomy for stage IB NSCLC (≤3 cm) with VPI, and wedge resection was associated with impaired survival. Adjuvant chemotherapy might not be associated with improved survival, even in those with sublobar resection. However, our findings might be interpreted with caution and require further validation in prospective controlled trials.

## Data Availability Statement

Publicly available datasets were analyzed in this study. This data can be found *via* SEER Stat (www.seer.cancer.gov) with the identifier 11151-Nov2019.

## Author Contributions

WH and H-YD contributed to the conception of the study and drafting of the article and took full responsibility for the content, including the data and analysis. WH, KX, M-YL, and Y-XZ contributed to the data extraction. WH, H-YD, M-YL, and CY contributed to the statistical analysis. WH, H-YD, and QZ contributed to the revision of the article. WH, H-YD, M-YL, KX, Y-XZ, CY, and QZ contributed to the approval of the final article.

## Conflict of Interest

The authors declare that the research was conducted in the absence of any commercial or financial relationships that could be construed as a potential conflict of interest.

## Publisher’s Note

All claims expressed in this article are solely those of the authors and do not necessarily represent those of their affiliated organizations, or those of the publisher, the editors and the reviewers. Any product that may be evaluated in this article, or claim that may be made by its manufacturer, is not guaranteed or endorsed by the publisher.

## Abbreviations

CI, confidence interval; CSS, cancer-specific survival; DFS, disease-free survival; HR, hazard ratio; LVI, lymphovascular invasion; NSCLC, non-small cell lung cancer; OS, overall survival; PL, pleural layer; PSM, propensity score matching; SEER, Surveillance, Epidemiology, and End Results; VPI, visceral pleural invasion.
