# Supplementary material for: Treatment Modality for Stage IB Peripheral Non-Small Cell Lung Cancer With Visceral Pleural Invasion and ≤3 cm in Size
Source: Front Oncol. 2022 Feb 18;12:830470. doi: 10.3389/fonc.2022.830470 (PMC8905598; doi:10.3389/fonc.2022.830470)
Supplement: Supplementary file 1 [file DataSheet_1.docx]

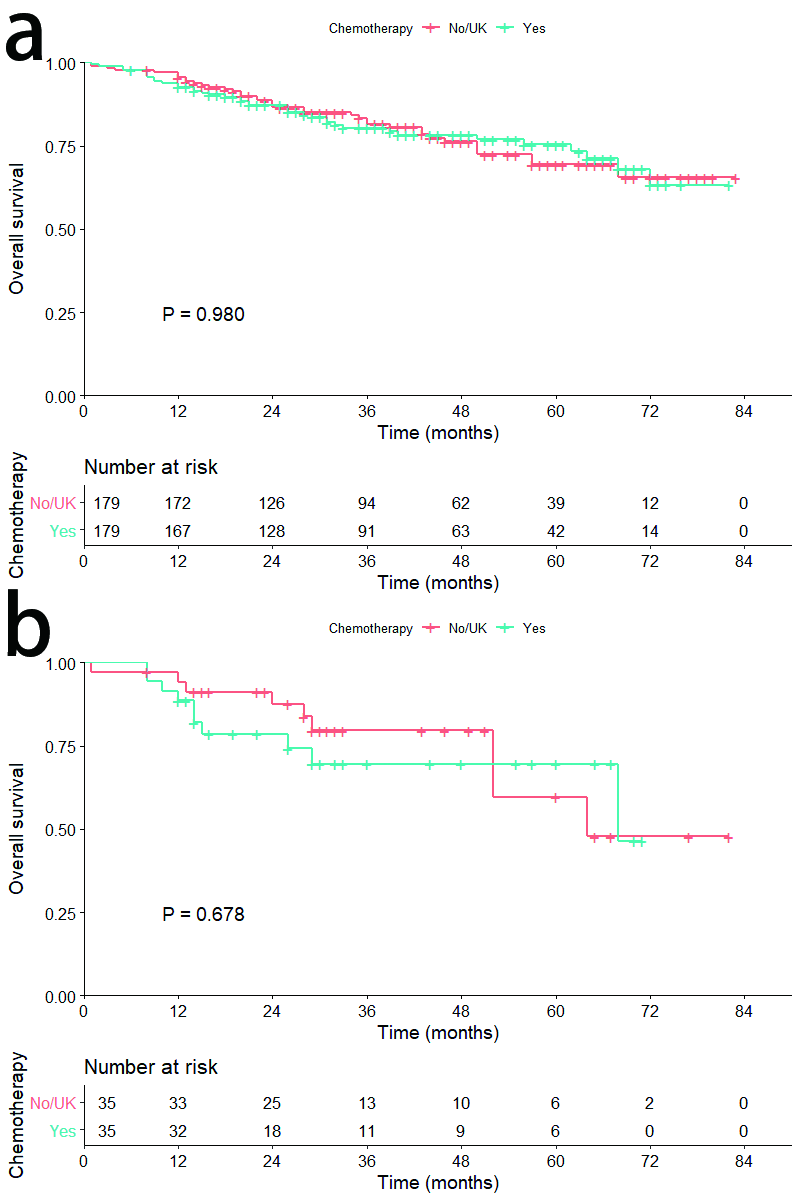


**e-Figure 1** Kaplan-Meier curves comparing the role of adjuvant chemotherapy in overall survival among the entire study population(a), and comparing the efficacy of adjuvant chemotherapy among those with sublobar resection in overall survival(b) after propensity score matching.

**e-Table 1** Demographics and baseline characteristics of the primary study population stratified by three surgical procedures before propensity score matching

| **Characteristics** | **Overall**  **(n=1993)** | **Lobectomy**  **(n=1420)** | **Segmentectomy**  **(n=116)** | **Wedge resection**  **(n=457)** | **P-Value** |
| --- | --- | --- | --- | --- | --- |
| Age |  |  |  |  | <0.001 |
| ≤70 years | 1033 (51.8) | 802 (56.5) | 50 (43.1) | 181 (39.6) |  |
| >70 years | 960 (48.2) | 618 (43.5) | 66 (56.9) | 276 (60.4) |  |
| Gender |  |  |  |  | 0.257 |
| Male | 899 (45.1) | 654 (46.1) | 45 (38.8) | 200 (43.8) |  |
| Female | 1094 (54.9) | 766 (53.9) | 71 (61.2) | 257 (56.2) |  |
| Race |  |  |  |  | 0.026 |
| White | 1651 (82.8) | 1152 (81.1) | 104 (89.7) | 395 (86.4) |  |
| Black | 196 (9.8) | 152 (10.7) | 7 (6.0) | 37 (8.1) |  |
| Other | 146 (7.3) | 116 (8.2) | 5 (4.3) | 25 (5.5) |  |
| Marital status |  |  |  |  | 0.217 |
| Married | 1074 (53.9) | 774 (54.5) | 68 (58.6) | 232 (50.8) |  |
| Other^a^ | 919 (46.1) | 646 (45.5) | 48 (41.4) | 225 (49.2) |  |
| Primary site |  |  |  |  | 0.003 |
| Lower lobe | 566 (28.4) | 393 (27.7) | 44 (37.9) | 129 (28.2) |  |
| Middle lobe | 152 (7.6) | 123 (8.7) | 0 (0.0) | 29 (6.3) |  |
| Upper lobe | 1275 (64.0) | 904 (63.7) | 72 (62.1) | 299 (65.4) |  |
| Laterality |  |  |  |  | 0.001 |
| Left | 823 (41.3) | 559 (39.4) | 66 (56.9) | 198 (43.3) |  |
| Right | 1170 (58.7) | 861 (60.6) | 50 (43.1) | 259 (56.7) |  |
| Tumor size |  |  |  |  | <0.001 |
| 1-10mm | 99 (5.0) | 53 (3.7) | 11 (9.5) | 35 (7.7) |  |
| 11-20mm | 935 (46.9) | 613 (43.2) | 58 (50.0) | 264 (57.8) |  |
| 21-30mm | 959 (48.1) | 754 (53.1) | 47 (40.5) | 158 (34.6) |  |
| Histology |  |  |  |  | 0.001 |
| AC | 1309 (65.7) | 960 (67.6) | 72 (62.1) | 277 (60.6) |  |
| SCC | 385 (19.3) | 241 (17.0) | 24 (20.7) | 120 (26.3) |  |
| Other | 299 (15.0) | 219 (15.4) | 20 (17.2) | 60 (13.1) |  |
| Grade^b^ |  |  |  |  | 0.160 |
| I | 235 (11.8) | 173 (12.2) | 13 (11.2) | 49 (10.7) |  |
| II | 1044 (52.4) | 759 (53.5) | 58 (50.0) | 227 (49.7) |  |
| III/IV | 629 (31.6) | 438 (30.8) | 37 (31.9) | 154 (33.7) |  |
| UK | 85 (4.3) | 50 (3.5) | 8 (6.9) | 27 (5.9) |  |
| LN examined^c^ |  |  |  |  | <0.001 |
| Yes | 1756 (88.1) | 1385 (97.5) | 91 (78.4) | 280 (61.3) |  |
| No/UK | 237 (11.9) | 35 (2.5) | 25 (21.6) | 177 (38.7) |  |
| Adjuvant treatment |  |  |  |  | 0.188 |
| Yes | 263 (13.2) | 180 (12.7) | 12 (10.3) | 71 (15.5) |  |
| No/UK | 1730 (86.8) | 1240 (87.3) | 104 (89.7) | 386 (84.5) |  |
| Radiotherapy |  |  |  |  | <0.001 |
| Yes | 80 (4.0) | 34 (2.4) | 5 (4.3) | 41 (9.0) |  |
| No/UK | 1913 (96.0) | 1386 (97.6) | 111 (95.7) | 416 (91.0) |  |
| Chemotherapy |  |  |  |  | 0.133 |
| Yes | 210 (10.5) | 162 (11.4) | 9 (7.8) | 39 (8.5) |  |
| No/UK | 1783 (89.5) | 1258 (88.6) | 107 (92.2) | 418 (91.5) |  |

^a^Other marital status included single, divorced, Unmarried or Domestic Partner, Separated, widowed, or unknown.

^b^Grade refers to the degree of cellular differentiation; I, well differentiated; II, moderately differentiated; III, poorly differentiated; IV, undifferentiated or anaplastic.

^c^Lymph nodes examined referred to the removal of regional lymph nodes, or biopsy of sentinel nodes with or without the removal of lymph nodes.

**Abbreviations:** AC, adenocarcinoma; SCC, squamous cell carcinoma; UK, unknown; LN, lymph node(s).

**e-Table 2** Demographics and baseline characteristics between lobectomy and sublobar resection after propensity score matching

| **Characteristics** | **Overall**  **(n=744)** | **Lobectomy**  **(n=372)** | **Sublobar resection**  **(n=372)** | **P-Value** |
| --- | --- | --- | --- | --- |
| Age |  |  |  | 0.600 |
| ≤70 years | 296 (39.8) | 152 (40.9) | 144 (38.7) |  |
| >70 years | 448 (60.2) | 220 (59.1) | 228 (61.3) |  |
| Gender |  |  |  | 0.882 |
| Male | 313 (42.1) | 155 (41.7) | 158 (42.5) |  |
| Female | 431 (57.9) | 217 (58.3) | 214 (57.5) |  |
| Race |  |  |  | 0.909 |
| White | 658 (88.4) | 330 (88.7) | 328 (88.2) |  |
| Other | 86 (11.6) | 42 (11.3) | 44 (11.8) |  |
| Marital status |  |  |  | 0.377 |
| Married | 409 (55.0) | 198 (53.2) | 211 (56.7) |  |
| Other | 335 (45.0) | 174 (46.8) | 161 (43.3) |  |
| Tumor size |  |  |  | 0.710 |
| 1-20mm | 436 (58.6) | 215 (57.8) | 221 (59.4) |  |
| 21-30mm | 308 (41.4) | 157 (42.2) | 151 (40.6) |  |
| Histology |  |  |  | 0.708 |
| AC | 450 (60.5) | 222 (59.7) | 228 (61.3) |  |
| SCC/Other | 294 (39.5) | 150 (40.3) | 144 (38.7) |  |
| Grade |  |  |  | 1.000 |
| I/II | 442 (59.4) | 221 (59.4) | 221 (59.4) |  |
| III/IV/UK | 302 (40.6) | 151 (40.6) | 151 (40.6) |  |
| LN examined |  |  |  | 0.798 |
| Yes | 677 (91.0) | 340 (91.4) | 337 (90.6) |  |
| No/UK | 67 (9.0) | 32 (8.6) | 35 (9.4) |  |
| Adjuvant treatment |  |  |  | 1.000 |
| Yes | 45 (6.0) | 22 (5.9) | 23 (6.2) |  |
| No/UK | 699 (94.0) | 350 (94.1) | 349 (93.8) |  |
| Chemotherapy |  |  |  | 1.000 |
| Yes | 45 (6.0) | 22 (5.9) | 23 (6.2) |  |
| No/UK | 699 (94.0) | 350 (94.1) | 349 (93.8) |  |

**Abbreviations:** AC, adenocarcinoma; SCC, squamous cell carcinoma; UK, unknown; LN, lymph node(s).

**e-Table 3** Demographics and baseline characteristics among the study population (excluded those with radiotherapy) grouped by the three surgical procedures after propensity score matching

| **Characteristics** | **Lobectomy versus Segmentectomy** | | |  | **Lobectomy versus Wedge resection** | | |  | **Segmentectomy versus Wedge resection** | | |
| --- | --- | --- | --- | --- | --- | --- | --- | --- | --- | --- | --- |
|  | **Lobectomy**  **(n=102)** | **Segmentectomy**  **(n=102)** | **P-Value** |  | **Lobectomy**  **(n=298)** | **Wedge resection**  **(n=298)** | **P-Value** |  | **Segmentectomy**  **(n=108)** | **Wedge resection**  **(n=108)** | **P-Value** |
| Age |  |  | 0.671 |  |  |  | 0.673 |  |  |  | 0.680 |
| ≤70 years | 41 (40.2) | 45 (44.1) |  |  | 116 (38.9) | 110 (36.9) |  |  | 44 (40.7) | 48 (44.4) |  |
| >70 | 61 (59.8) | 57 (55.9) |  |  | 182 (61.1) | 188 (63.1) |  |  | 64 (59.3) | 60 (55.6) |  |
| Gender |  |  | 0.774 |  |  |  | 0.680 |  |  |  | 0.574 |
| Male | 41 (40.2) | 38 (37.3) |  |  | 129 (43.3) | 135 (45.3) |  |  | 43 (39.8) | 38 (35.2) |  |
| Female | 61 (59.8) | 64 (62.7) |  |  | 169 (56.7) | 163 (54.7) |  |  | 65 (60.2) | 70 (64.8) |  |
| Race |  |  | 1.000 |  |  |  | 0.514 |  |  |  | 1.000 |
| White | 92 (90.2) | 93 (91.2) |  |  | 268 (89.9) | 262 (87.9) |  |  | 97 (89.8) | 97 (89.8) |  |
| Other | 10 (9.8) | 9 (8.8) |  |  | 30 (10.1) | 36 (12.1) |  |  | 11 (10.2) | 11 (10.2) |  |
| Marital status |  |  | 0.888 |  |  |  | 0.620 |  |  |  | 1.000 |
| Married | 56 (54.9) | 58 (56.9) |  |  | 173 (58.1) | 166 (55.7) |  |  | 62 (57.4) | 63 (58.3) |  |
| Other | 46 (45.1) | 44 (43.1) |  |  | 125 (41.9) | 132 (44.3) |  |  | 46 (42.6) | 45 (41.7) |  |
| Tumor size |  |  | 0.671 |  |  |  | 1.000 |  |  |  | 0.779 |
| 1-20mm | 57 (55.9) | 61 (59.8) |  |  | 186 (62.4) | 186 (62.4) |  |  | 66 (61.1) | 69 (63.9) |  |
| 21-30mm | 45 (44.1) | 41 (40.2) |  |  | 112 (37.6) | 112 (37.6) |  |  | 42 (38.9) | 39 (36.1) |  |
| Histology |  |  | 0.202 |  |  |  | 0.803 |  |  |  | 1.000 |
| AC | 54 (52.9) | 64 (62.7) |  |  | 174 (58.4) | 178 (59.7) |  |  | 66 (61.1) | 66 (61.1) |  |
| SCC/Other | 48 (47.1) | 38 (37.3) |  |  | 124 (41.6) | 120 (40.3) |  |  | 42 (38.9) | 42 (38.9) |  |
| Grade |  |  | 0.384 |  |  |  | 1.000 |  |  |  | 0.779 |
| I/II | 68 (66.7) | 61 (59.8) |  |  | 178 (59.7) | 178 (59.7) |  |  | 66 (61.1) | 69 (63.9) |  |
| III/IV/UK | 34 (33.3) | 41 (40.2) |  |  | 120 (40.3) | 120 (40.3) |  |  | 42 (38.9) | 39 (36.1) |  |
| LN examined |  |  | 0.843 |  |  |  | 0.609 |  |  |  | 0.874 |
| Yes | 88 (86.3) | 86 (84.3) |  |  | 266 (89.3) | 261 (87.6) |  |  | 83 (76.9) | 81 (75.0) |  |
| No/UK | 14 (13.7) | 16 (15.7) |  |  | 32 (10.7) | 37 (12.4) |  |  | 25 (23.1) | 27 (25.0) |  |
| Chemotherapy |  |  | 0.766 |  |  |  | 0.486 |  |  |  | 1.000 |
| Yes | 5 (4.9) | 7 (6.9) |  |  | 15 (5.0) | 20 (6.7) |  |  | 6 (5.6) | 5 (4.6) |  |
| No/UK | 97 (95.1) | 95 (93.1) |  |  | 283 (95.0) | 278 (93.3) |  |  | 102 (94.4) | 103 (95.4) |  |

**Abbreviations:** AC, adenocarcinoma; SCC, squamous cell carcinoma; UK, unknown; LN, lymph node(s).

**e-Table 4** Subgroup analyses comparing the overall survival among the study population grouped by the three surgical procedures via cox regression analysis after propensity score matching

| **Characteristics** | **Segmentectomy versus Lobectomy** | |  | **Wedge resection versus Lobectomy** | |  | **Wedge resection versus Segmentectomy** | |
| --- | --- | --- | --- | --- | --- | --- | --- | --- |
|  | **HR (95%CI)** | **P-Value** |  | **HR (95%CI)** | **P-Value** |  | **HR (95%CI)** | **P-Value** |
| Age |  |  |  |  |  |  |  |  |
| ≤70 years | 1.06 (0.46-2.46) | 0.891 |  | 1.39 (0.81-2.36) | 0.228 |  | 0.79 (0.36-1.73) | 0.550 |
| >70 years | 1.22 (0.60-2.51) | 0.585 |  | 1.18 (0.85-1.63) | 0.317 |  | 1.27 (0.72-2.25) | 0.409 |
| Gender |  |  |  |  |  |  |  |  |
| Male | 1.02 (0.46-2.27) | 0.959 |  | 1.15 (0.77-1.70) | 0.501 |  | 0.90 (0.42-1.93) | 0.789 |
| Female | 1.18 (0.59-2.33) | 0.643 |  | 1.54 (1.02-2.31) | 0.040 |  | 1.28 (0.72-2.27) | 0.394 |
| Tumor size |  |  |  |  |  |  |  |  |
| 1-20mm | 0.79 (0.38-1.64) | 0.527 |  | 1.18 (0.81-1.72) | 0.396 |  | 0.90 (0.47-1.71) | 0.743 |
| 21-30mm | 1.49 (0.63-3.48) | 0.362 |  | 1.30 (0.85-1.98) | 0.225 |  | 1.09 (0.55-2.16) | 0.807 |
| Histology |  |  |  |  |  |  |  |  |
| AC | 1.00 (0.52-1.95) | 0.996 |  | 1.56 (1.06-2.29) | 0.023 |  | 0.73 (0.38-1.41) | 0.349 |
| SCC/Others | 1.25 (0.58-2.73) | 0.569 |  | 1.30 (0.84-2.01) | 0.238 |  | 1.36 (0.66-2.84) | 0.405 |
| Grade |  |  |  |  |  |  |  |  |
| I/II | 1.25 (0.61-2.54) | 0.543 |  | 1.40 (0.97-2.02) | 0.075 |  | 1.08 (0.58-2.02) | 0.804 |
| III/IV/UK | 0.55 (0.26-1.15) | 0.112 |  | 1.36 (0.89-2.09) | 0.154 |  | 1.11 (0.54-2.27) | 0.783 |

**Abbreviations:** HR, hazard ratio; CI, confidence interval; AC, adenocarcinoma; SCC, squamous cell carcinoma; UK, unknown; LN, lymph node(s).

**e-Table 5** Demographics and baseline characteristics among those over 70 years after propensity score matching regarding the three surgical procedures

| **Characteristics** | **Lobectomy versus Segmentectomy** | | |  | **Lobectomy versus Wedge resection** | | |  | **Segmentectomy versus Wedge resection** | | |
| --- | --- | --- | --- | --- | --- | --- | --- | --- | --- | --- | --- |
|  | **Lobectomy**  **(n=54)** | **Segmentectomy**  **(n=54)** | **P-Value** |  | **Lobectomy**  **(n=182)** | **Wedge resection**  **(n=182)** | **P-Value** |  | **Segmentectomy**  **(n=63)** | **Wedge resection**  **(n=63)** | **P-Value** |
| Gender |  |  | 0.441 |  |  |  | 0.833 |  |  |  | 0.476 |
| Male | 28 (51.9) | 23 (42.6) |  |  | 82 (45.1) | 85 (46.7) |  |  | 30 (47.6) | 35 (55.6) |  |
| Female | 26 (48.1) | 31 (57.4) |  |  | 100 (54.9) | 97 (53.3) |  |  | 33 (52.4) | 28 (44.4) |  |
| Race |  |  | 0.486 |  |  |  | 0.861 |  |  |  | 1.000 |
| White | 48 (88.9) | 51 (94.4) |  |  | 165 (90.7) | 163 (89.6) |  |  | 59 (93.7) | 59 (93.7) |  |
| Other | 6 (11.1) | 3 (5.6) |  |  | 17 (9.3) | 19 (10.4) |  |  | 4 (6.3) | 4 (6.3) |  |
| Marital status |  |  | 0.846 |  |  |  | 0.752 |  |  |  | 0.856 |
| Married | 32 (59.3) | 30 (55.6) |  |  | 96 (52.7) | 100 (54.9) |  |  | 37 (58.7) | 39 (61.9) |  |
| Other | 22 (40.7) | 24 (44.4) |  |  | 86 (47.3) | 82 (45.1) |  |  | 26 (41.3) | 24 (38.1) |  |
| Tumor size |  |  | 0.699 |  |  |  | 0.748 |  |  |  | 1.000 |
| 1-20mm | 28 (51.9) | 31 (57.4) |  |  | 112 (61.5) | 108 (59.3) |  |  | 36 (57.1) | 37 (58.7) |  |
| 21-30mm | 26 (48.1) | 23 (42.6) |  |  | 70 (38.5) | 74 (40.7) |  |  | 27 (42.9) | 26 (41.3) |  |
| Histology |  |  | 0.336 |  |  |  | 0.916 |  |  |  | 0.858 |
| AC | 24 (44.4) | 30 (55.6) |  |  | 103 (56.6) | 101 (55.5) |  |  | 35 (55.6) | 33 (52.4) |  |
| SCC/Other | 30 (55.6) | 24 (44.4) |  |  | 79 (43.4) | 81 (44.5) |  |  | 28 (44.4) | 30 (47.6) |  |
| Grade |  |  | 0.559 |  |  |  | 0.832 |  |  |  | 1.000 |
| I/II | 29 (53.7) | 33 (61.1) |  |  | 108 (59.3) | 105 (57.7) |  |  | 38 (60.3) | 39 (61.9) |  |
| III/IV/UK | 25 (46.3) | 21 (38.9) |  |  | 74 (40.7) | 77 (42.3) |  |  | 25 (39.7) | 24 (38.1) |  |
| LN examined |  |  | 1.000 |  |  |  | 1.000 |  |  |  | 1.000 |
| Yes | 51 (94.4) | 51 (94.4) |  |  | 166 (91.2) | 165 (90.7) |  |  | 50 (79.4) | 49 (77.8) |  |
| No/UK | 3 (5.6) | 3 (5.6) |  |  | 16 (8.8) | 17 (9.3) |  |  | 13 (20.6) | 14 (22.2) |  |
| Chemotherapy |  |  | 1.000 |  |  |  | 0.414 |  |  |  | 1.000 |
| Yes | 1 (1.9) | 2 (3.7) |  |  | 9 (4.9) | 5 (2.7) |  |  | 2 (3.2) | 2 (3.2) |  |
| No/UK | 53 (98.1) | 52 (96.3) |  |  | 173 (95.1) | 177 (97.3) |  |  | 61 (96.8) | 61 (96.8) |  |

**Abbreviations:** AC, adenocarcinoma; SCC, squamous cell carcinoma; UK, unknown; LN, lymph node(s).

**e-Table 6** Subgroup analysis comparing three surgical procedures among those over 70 years in overall survival via cox regression analysis after propensity score matching

| **Characteristics** | **Segmentectomy versus lobectomy** | |  | **Wedge resection versus lobectomy** | |  | **Wedge resection versus segmentectomy** | |
| --- | --- | --- | --- | --- | --- | --- | --- | --- |
|  | **HR (95%CI)** | **P-Value** |  | **HR (95%CI)** | **P-Value** |  | **HR (95%CI)** | **P-Value** |
| Gender |  |  |  |  |  |  |  |  |
| Male | 0.56 (0.19-1.65) | 0.292 |  | 0.91 (0.56-1.47) | 0.691 |  | 1.39 (0.61-3.19) | 0.437 |
| Female | 1.62 (0.59-4.47) | 0.352 |  | 1.57 (0.98-2.51) | 0.059 |  | 0.66 (0.26-1.69) | 0.391 |
| Tumor size |  |  |  |  |  |  |  |  |
| 1-20mm | 0.81 (0.32-2.02) | 0.646 |  | 1.49 (0.94-2.34) | 0.087 |  | 1.09 (0.47-2.51) | 0.838 |
| 21-30mm | 1.44 (0.48-4.3) | 0.513 |  | 0.96 (0.58-1.59) | 0.871 |  | 0.99 (0.42-2.33) | 0.975 |
| Histology |  |  |  |  |  |  |  |  |
| AC | 1.2 (0.43-3.32) | 0.728 |  | 1.49 (0.93-2.39) | 0.097 |  | 0.96 (0.42-2.23) | 0.932 |
| SCC/Other | 0.85 (0.31-2.28) | 0.741 |  | 1.01 (0.63-1.64) | 0.964 |  | 1.09 (0.46-2.58) | 0.842 |
| Grade |  |  |  |  |  |  |  |  |
| I/II | 1.03 (0.42-2.55) | 0.943 |  | 1.01 (0.66-1.57) | 0.949 |  | 0.73 (0.32-1.68) | 0.462 |
| III/IV/UK | 0.99 (0.33-2.95) | 0.989 |  | 1.6 (0.94-2.7) | 0.081 |  | 1.59 (0.67-3.77) | 0.297 |

**Abbreviations:** HR, hazard ratio; CI, confidence interval; AC, adenocarcinoma; SCC, squamous cell carcinoma; UK, unknown.

**e-Table 7** Demographics and baseline characteristics among the patients with or without adjuvant chemotherapy after propensity score matching

| **Characteristics** | **Overall**  **(n=358)** | **No/UK**  **(n=179)** | **Yes**  **(n=179)** | **P-Value** |
| --- | --- | --- | --- | --- |
| Age |  |  |  | 0.473 |
| ≤70 years | 263 (73.5) | 128 (71.5) | 135 (75.4) |  |
| >70 years | 95 (26.5) | 51 (28.5) | 44 (24.6) |  |
| Gender |  |  |  | 0.084 |
| Male | 141 (39.4) | 62 (34.6) | 79 (44.1) |  |
| Female | 217 (60.6) | 117 (65.4) | 100 (55.9) |  |
| Race |  |  |  | 0.588 |
| White | 291 (81.3) | 148 (82.7) | 143 (79.9) |  |
| Other | 67 (18.7) | 31 (17.3) | 36 (20.1) |  |
| Marital status |  |  |  | 0.341 |
| Married | 186 (52.0) | 88 (49.2) | 98 (54.7) |  |
| Other | 172 (48.0) | 91 (50.8) | 81 (45.3) |  |
| Tumor size |  |  |  | 0.398 |
| 1-20mm | 179 (50.0) | 85 (47.5) | 94 (52.5) |  |
| 21-30mm | 179 (50.0) | 94 (52.5) | 85 (47.5) |  |
| Histology |  |  |  | 0.400 |
| AC | 264 (73.7) | 136 (76.0) | 128 (71.5) |  |
| SCC/Other | 94 (26.3) | 43 (24.0) | 51 (28.5) |  |
| Grade |  |  |  | 0.668 |
| I/II | 209 (58.4) | 102 (57.0) | 107 (59.8) |  |
| III/IV/UK | 149 (41.6) | 77 (43.0) | 72 (40.2) |  |
| Surgery |  |  |  | 1.000 |
| Lobectomy | 286 (79.9) | 143 (79.9) | 143 (79.9) |  |
| Sublobar resection | 72 (20.1) | 36 (20.1) | 36 (20.1) |  |
| LN examined |  |  |  | 0.849 |
| Yes | 328 (91.6) | 165 (92.2) | 163 (91.1) |  |
| No/UK | 30 (8.4) | 14 (7.8) | 16 (8.9) |  |

**Abbreviations:** AC, adenocarcinoma; SCC, squamous cell carcinoma; UK, unknown; LN, lymph node(s).

**e-Table 8** Demographics and baseline characteristics in those with sublobar resection after propensity score matching of receiving adjuvant chemotherapy

| **Characteristics** | **Overall**  **(n=70)** | **No chemotherapy**  **(n=35)** | **Received chemotherapy**  **(n=35)** | **P-Value** |
| --- | --- | --- | --- | --- |
| Age |  |  |  | 1.000 |
| ≤70 | 47 (67.1) | 23 (65.7) | 24 (68.6) |  |
| >70 | 23 (32.9) | 12 (34.3) | 11 (31.4) |  |
| Gender |  |  |  | 0.811 |
| Male | 34 (48.6) | 16 (45.7) | 18 (51.4) |  |
| Female | 36 (51.4) | 19 (54.3) | 17 (48.6) |  |
| Race |  |  |  | 0.081 |
| White | 55 (78.6) | 24 (68.6) | 31 (88.6) |  |
| Other | 15 (21.4) | 11 (31.4) | 4 (11.4) |  |
| Marital status |  |  |  | 0.809 |
| Married | 40 (57.1) | 21 (60.0) | 19 (54.3) |  |
| Other | 30 (42.9) | 14 (40.0) | 16 (45.7) |  |
| Tumor size |  |  |  | 0.450 |
| 1-20mm | 46 (65.7) | 21 (60.0) | 25 (71.4) |  |
| 21-30mm | 24 (34.3) | 14 (40.0) | 10 (28.6) |  |
| Histology |  |  |  | 0.810 |
| AC | 38 (54.3) | 18 (51.4) | 20 (57.1) |  |
| SCC/Other | 32 (45.7) | 17 (48.6) | 15 (42.9) |  |
| Grade |  |  |  | 0.805 |
| I/II | 44 (62.9) | 23 (65.7) | 21 (60.0) |  |
| III/IV/UK | 26 (37.1) | 12 (34.3) | 14 (40.0) |  |
| Surgery |  |  |  | 0.776 |
| Segmentectomy | 16 (22.9) | 9 (25.7) | 7 (20.0) |  |
| Wedge resection | 54 (77.1) | 26 (74.3) | 28 (80.0) |  |
| LN examined |  |  |  | 0.309 |
| Yes | 47 (67.1) | 26 (74.3) | 21 (60.0) |  |
| No/UK | 23 (32.9) | 9 (25.7) | 14 (40.0) |  |

**Abbreviations:** AC, adenocarcinoma; SCC, squamous cell carcinoma; UK, unknown; LN, lymph node(s)
